# A Standardised Method to Quantify the Infectious Titre of Rabbit Haemorrhagic Disease Virus

**DOI:** 10.3390/v17050609

**Published:** 2025-04-24

**Authors:** Tiffany W. O’Connor, Damian Collins, Andrew J. Read, Paul M. Hick, Peter D. Kirkland

**Affiliations:** Elizabeth Macarthur Agricultural Institute, NSW Department of Primary Industries and Regional Development, Menangle, NSW 2568, Australiadamian.collins@dpird.nsw.gov.au (D.C.); andrew.j.read@dpi.nsw.gov.au (A.J.R.); paul.hick@dpi.nsw.gov.au (P.M.H.)

**Keywords:** RHDV, virus infectivity, virus titration, bioassay

## Abstract

Quantifying the infectious titre of preparations containing rabbit haemorrhagic disease virus (RHDV) is an essential virological technique during RHDV research. The infectious titre of an RHDV preparation is determined using a bioassay to identify the endpoint dilution at which 50% of rabbits become infected (RID_50_). Previous publications have briefly described the method for estimating the infectious titre of RHDV preparations by challenging rabbits with 10-fold serial dilutions. However, these descriptions lack the critical considerations for a standardised method to estimate RID_50_. These details are presented here, along with a comparison between the Reed–Muench, Dragstedt–Behrens, Spearman–Kärber, and probit regression methods for calculating the RID_50_. All the statistical approaches demonstrated a high level of agreement in calculating the RID_50_. To help assess the precision of the estimated infectious titre, the improved Spearman–Kärber and probit regression methods provide the 95% confidence intervals. The method outlined improves the accuracy of results when undertaking studies of pathogenicity, host resistance, and the production of vaccines against RHDV.

## 1. Introduction

Globally, rabbit haemorrhagic disease virus (RHDV; genus *Lagovirus*, family *Caliciviridae*) is an important pathogen of lagomorph populations. The emergence of genotype GI.2 (RHDV2) has resulted in the increase in the host range of RHDV from European rabbits (*Oryctolagus cuniculus*) to wild populations of Antelope rabbits (*Lepus alleni*), Desert cottontails (*Sylvilagus audubonii),* Mountain cottontails (*Sylvilagus nuttallii*), and Eastern cottontails (*Sylvilagus floridanus*) [1,2]. Unlike RHDV GI.1 (RHDV1), RHDV2 also causes disease in hares (*Lepus capensis var. mediterraneus, Lepus corsicanus, Lepus europaeus,* and *Lepus timidus*) [3,4,5,6]. Research has been undertaken to compare the virulence of RHDV strains [7,8,9], assess host resistance [2,10], and evaluate vaccines [6].

The multiplication of RHDV cannot be achieved in vitro using cell cultures; each of these investigations is heavily dependent on both the in vivo replication of the virus in rabbits and subsequent quantification using an in vivo bioassay based on the inoculation of rabbits. RHDV preparations used in experimental studies often consist of liver homogenates with different levels of purification and of varying concentrations [7]. Differences in the quality of a preparation, susceptibility of rabbits used in a bioassay, and methods used to quantify the titre of infectious virus can all impact on experimental outcomes, with significant variations observed even within single studies [7]. Furthermore, comparisons between studies are difficult when the methods used to quantify the amount of virus used have not been calculated on the basis of infectivity [7].

Methods that estimate RNA or antigen concentrations are unlikely to provide an indication of the amount of infectious virus in an inoculum with sufficient precision to give a reliable outcome. These assays cannot distinguish between viable virus and non-infectious viral components [11,12]. While RHDV frequently causes death, the lethal dose may not provide a valid estimate of infectivity as the occurrence of death is influenced by the virulence and pathogenicity of a strain of virus and host factors that influence pathogenicity [13,14]. Additionally, incomplete or defective virus particles that lack infectivity can lead to differences between RNA and antigen concentrations and infectivity [12].

For RHDV, the infectious unit is expressed as the median rabbit infectious dose (RID_50_), which represents the endpoint dilution at which 50% of rabbits become infected [8,15]. The titre of an RHDV preparation is expressed as the number of infectious units in a specified volume, usually as RID_50_ per mL.

Quantifying the infectious titre of an RHDV preparation involves the inoculation of groups of rabbits with serial dilutions of the preparation in question. Only two peer-reviewed papers describe the titration of RHDV in rabbits [8,15]. Both publications provide a single-sentence description of the experimental method: “For titration, groups of six adult rabbits were inoculated intramuscularly with 10-fold serial dilutions of the concentrated virus stock, and the Reed-Muench method was used to determine the 50% endpoint”. However, factors that could impact on RHDV replication in rabbits are missing from these descriptions and could affect the repeatability and reproducibility for estimates of the RID_50_ from the bioassay. The virus titre (expressed as RID_50_/mL) can be calculated using interpolation or curve-fitting methods, determined from the number of infected and non-infected rabbits in the bioassay [16,17]. The Reed–Muench (RM) method has been used frequently to quantify RHDV preparations [8,15,18] by interpolation between two doses that have endpoints based on counts above and below 50% [16]. Alternative approaches using interpolation include the Dragstedt–Behrens (DB) [19] and Spearman–Kärber (SK) methods [20,21]. Mathematically, the improved SK method may be the most reliable [17]. In addition, the estimates for the 50% endpoint across all three interpolation methods are consistent and can be calculated using spreadsheets or even manually [22,23]. In contrast, curve-fitting methods, like probit regression, have seen limited use in virology, partly due to the challenges in implementing this statistical approach [24,25,26,27]. To address accessibility, we have developed an online calculator for the probit regression method to enable researchers to input data and obtain RID_50_ estimates without specialised statistical software (https://virus-shiny-data.shinyapps.io/RID50/, accessed on 28 February 2025). Similar to the improved SK method, probit regression also provides confidence limits for the estimate which facilitates the evaluation of the precision of the calculated RID_50_.

In order to ensure that estimates of RID_50_ are accurate and repeatable there are a number of essential considerations, including rabbit selection and the absence of immunity to RHDV, inoculum preparation, infection control measures, the duration of the bioassay, criteria used to define infection status, and the statistical method (s) used for calculations. The objectives of this study were to (1) describe a standardised method for estimating RID_50_ and (2) compare the statistical methods used to calculate RID_50_ including approaches that can provide a measure of precision.

## 2. Materials and Methods

### 2.1. Rabbit Selection and Housing

New Zealand white unvaccinated rabbits of either sex, older than 12 weeks, and free of antibodies to all strains of RHDV are selected for the bioassay. Blood samples are collected before selection and tested for RHDV antibodies using the three ELISAs previously described for RHDV1, RHDV2, and RHDV GI.4 (RCV-A1) [28]. A minimum of 28 rabbits should be selected and assigned at random to one of 4 groups to provide at least 6 rabbits for each dose of diluted RHDV inoculum and one uninfected control to be co-housed with each group.

For the duration of the bioassay, the rabbits are housed in insect-proof biological containment, following the guidelines stipulated for the care and management of rabbits in scientific institutions [29] and the protocol approved by the institute’s Animal Ethics Committee. The rabbits are transferred to housing that segregates each rabbit in a manner that prevents direct contact and short distance aerosol transmission between individual rabbits and between groups. Practically, this is achieved by placing seven appropriately sized cages in a separate room for groups of rabbits inoculated with each dilution and the unchallenged control. They are acclimated for at least 24 h before inoculation.

An RHDV inoculum usually consists of homogenates of infected rabbit livers, which can be prepared at varying levels of concentration or purification [30,31]. An estimation of the range of dilutions to be prepared is based on past experience and may be guided by hemagglutination or qRT-PCR. At least four ten-fold dilutions should be prepared using sterile phosphate-buffered saline (pH 7.2) as the diluent. The dilution with the highest concentration of virus should be 1 log_10_ higher than the predicted 50% endpoint with 3 additional dilutions prepared to give lower virus concentrations. The accuracy of the dilution series is confirmed using a reverse-transcription, real-time PCR (RT-qPCR) specific to RHDV1 [32] or RHDV2 [33] depending on the virus used as the inoculum.

The rabbits are given an intramuscular injection with 1 mL of the diluted RHDV inoculum and observed 2–3 times daily for seven days, after which the bioassay is terminated by sedation prior to euthanasia to ensure that the infection status of each rabbit can be determined.

Following the bioassay’s conclusion, all consumables are autoclaved and discarded to prevent the potential carryover of infectious RHDV between experiments. The equipment is cleaned and soaked in a suitable disinfectant (e.g., 1% F10SC Veterinary Disinfectant, Health and Hygiene, South Africa) for at least 18 h. The animal holding facility is also cleaned and disinfected in a similar manner by removing all organic matter, cleaning and applying a disinfectant spray, and then leaving for the relevant contact time. Finally, after all the equipment and the facility are dry, the room and remaining equipment are fumigated (e.g., paraformaldehyde prilled, Merck Life Science, Melbourne, Australia) to ensure thorough decontamination.

### 2.2. Criteria Used to Determine Infection Status

Seven days after inoculation, liver samples are collected from each rabbit. The liver is exposed by making a ventral midline incision with a sterile scalpel. A second sterile scalpel is used to incise the surface of the liver across multiple lobes. A sterile dry swab is passed vigorously across the freshly cut liver surfaces until saturated and placed into 3 mL of sterile phosphate-buffered saline (pH 7.2) supplemented with 0.5% gelatin (PBGS). The PBGS from each rabbit is tested by RT-qPCR to detect RHDV RNA [32,33].

A rabbit is classified as RHDV-infected if there is evidence of viral replication. It is considered that virus replication has occurred when the cycle-threshold (Ct) value from the liver swab is less than the Ct value of the inoculum administered to the rabbit. A rabbit was classified as not-infected if virus replication is not detected.

### 2.3. Calculating the Median Rabbit Infectious Dose and Infectious Titre of the RHDV Inoculum

For each dose of diluted RHDV inoculum, the count of RHDV-infected and non-infected rabbits is used to construct the dose–response relationship and calculate the infectious virus load from the RID_50_. An example of the statistical analyses used to calculate RID_50_, is provided in Appendix A to illustrate the RM [18], DB [19], SK [20,21], and probit regression [24,25] methods. The analyses were performed in R (v4.4.0; R Core Team, 2024 [34]) using the *skrmdb* (version 4.5.0) and the *MASS* (version 7.3-60.2) packages [35,36]. To remove the need for statistical software to complete the probit regression, an online calculator including the profile likelihood (PL) function for the 95% confidence interval was developed using Shiny [37]. This calculator is available at https://virus-shiny-data.shinyapps.io/RID50/. After taking into account the dilution factor at the chosen endpoint, the infectious titre of the RHDV preparation is calculated and is expressed as RID_50_ per mL.

### 2.4. Assessing the Agreement Between the Calculation Approaches for RID_50_

The agreement between the analyses used to calculate RID_50_ was evaluated using data from 26 separate bioassays undertaken to quantify the infectious titre of different preparations of RHDV. The calculated RID_50_ from the RM, DB, SK, and probit regression methods were visualised using the Bland–Altman plot [38] and assessed using the intra-class correlation coefficient (ICC) calculated using the *irr* (version 0.84.1) package in R [39]. A two-way random-effects model was selected with an absolute agreement definition. The ICC results were interpreted as follows: values below 0.50 indicate poor agreement, 0.50–0.75 indicate moderate agreement, 0.75–0.90 indicate good agreement, and values above 0.90 indicate excellent agreement [40].

## 3. Results

**Figure 1:**
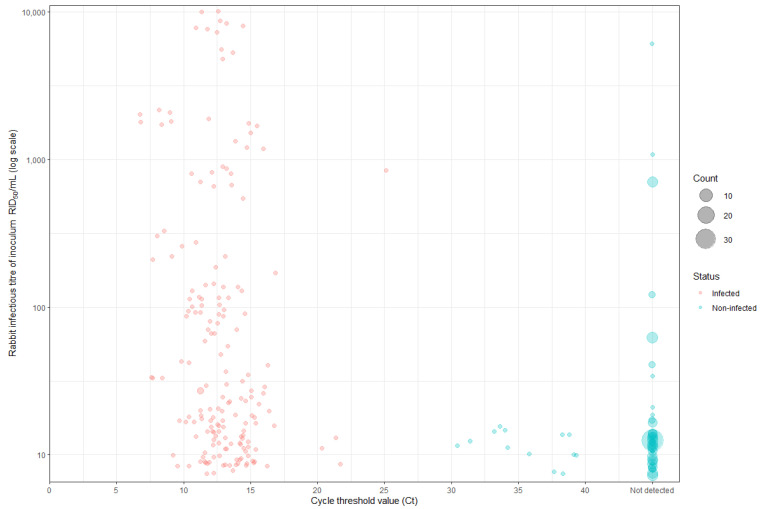
Distribution of cycle threshold (Ct) values from liver swabs collected from all the rabbits in the bioassays to determine the infectious titre (RID_50_/mL) of rabbit haemorrhagic disease virus (RHDV) preparations. The RID_50_/mL values, depicted on a log_10_ scale, were calculated using the Reed–Muench method. Individual data points represent single rabbits, with RHDV-infected rabbits in red and non-infected rabbits in blue. The point size also corresponds to the number of rabbits that received the same dose and had the same RT-qPCR result.

From the 26 bioassays, a total of 542 rabbits were challenged with doses in a ten-fold dilution series from 10^−2^ to 10^−8^ (all <10,000 RID_50_/mL, Figure 1). An average of six rabbits were inoculated per dose (min. 4, max. 9) across at least four dilutions, typically from 10^−3^ to 10^−6^.

Liver swabs from RHDV-infected rabbits had a range of 6.74 to 25.1 (Q1 11.4, median 12.7 and Q3 14.3, Figure 1). The Ct difference between the inoculum and the liver swab from RHDV-infected rabbits ranged from 3.7 to 22.6 (Q1 9.1, median 12.1 and Q3 15.6).

RHDV RNA was detected in non-infected rabbits on 13 occasions (5.7%, out of 230 non-infected rabbits). For these 13 non-infected rabbits, the liver swabs ranged from 30.5 to 39.3 (Q1 33.6, median 35.84 and Q3 38.4). The Ct values for liver swabs from non-infected rabbits were higher than those of the inoculum by a range of 0.64 to 6.9 cycles (Q1 2.9, median 3.0 and Q3 3.3).

**Figure 2:**
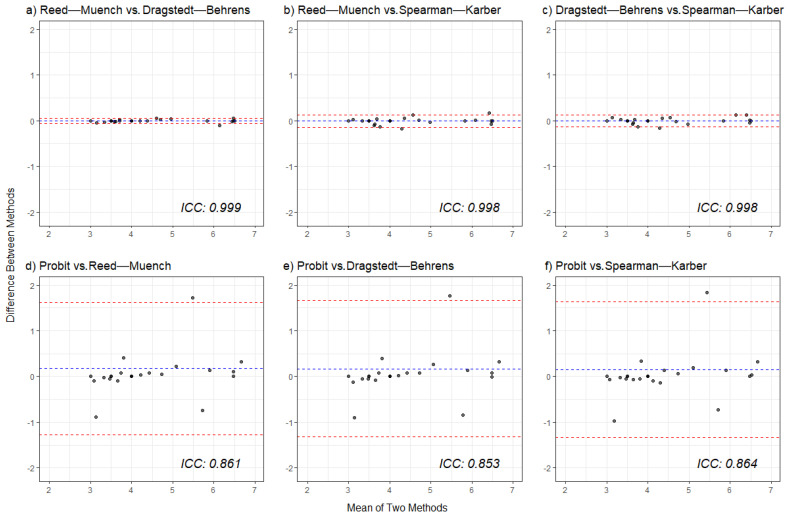
Bland–Altman plots comparing the agreement between different methods to calculate the median rabbit infectious dose (RID_50_). Each plot (**a**–**f**) displays the difference between the two methods against their mean, with the blue dashed line representing the mean difference and the red dashed lines indicating the 95% limits of agreement. The intraclass correlation coefficient (ICC) for each pair is displayed in each plot.

The results from the 26 bioassays were used to compare the statistical approaches used to calculate the infectious titre of RHDV (Table 1; Supplementary Figure S1). The three interpolative methods approached complete agreement: RM and DB (ICC = 0.999), RM and SK (ICC = 0.998), and DB and SK (ICC = 0.998) (Figure 2a–c).

The ICC between probit and RM (ICC = 0.861), probit and DB (ICC = 0.853), and probit and SK (ICC = 0.864) methods fall within the good agreement range (Figure 2d–f). The Bland–Altman plots support this, showing mean differences close to zero with most data points within the 95% limits of agreement.

## 4. Discussion

Accurately quantifying the infectious titre of an RHDV preparation is crucial to ensure that a consistent viral dose is used in experimental studies undertaken to compare virulence of strains, assess host resistance or to demonstrate vaccine efficacy. A bioassay that provides a repeatable and reproducible measure of the RID_50_ is essential.

*Selection of rabbits*

Rabbits need to be over 12 weeks of age as younger rabbits are not susceptible to RHDV1 infection [41,42,43]. To ensure susceptibility, all the rabbits should be tested for RHDV antibodies and demonstrated to be seronegative for all genogroups. Pre-existing immunity from vaccination, natural exposure or maternal antibodies will impact infection outcomes [28,44,45]. Further, the breed and strain of rabbits is important as genetic resistance to infection with RHDV has been reported in some rabbit populations [9,46,47]. Typically, laboratory-bred New Zealand white rabbits are used, although other albino breeds may be suitable [7,8,28]. Albino rabbits are preferred because melatonin could provide some protective effects against RHDV infection [48]. Rabbits from a source farm with potential selection for resistance to RHDV infection should be avoided.

*Inoculum preparation*

The infectious titre must be quantified from an aliquot stored in the same manner as the material intended for experimental investigation. Repeated freezing and thawing can significantly reduce the infectious titre of RHDV [49]. The range of dilutions tested must to extend across the entire dose–response curve from 100% of rabbits being infected at the lowest dilution to no evidence of infection at the highest dilution [16]. The accuracy of the 10-fold dilution series is confirmed prior to inoculation by quantifying RHDV RNA by RT-qPCR [50], whereby each log_10_ dilution should result in an increase in Ct value of approximately 3.3 [51]. A dilution series is considered suitable if the Ct values increase by 3.3 ± 0.5.

*Bioassay conditions*

Infection control measures in the animal housing facility are important to ensure that there is no extraneous source of RHDV, minimising the risk of rabbits being exposed to a challenge that is different from the intended dose of inoculum, or cross-contamination during sample collection. Additionally, rabbits are protected from sources of RHDV infection from fomites and vectors external to the study. Natural transmission of RHDV generally occurs by direct contact with an infected animal, indirectly on fomites such as feed, bedding, cages and equipment, or by mechanical transmission by flying insects [52,53]. Rabbits are housed individually in insect-proof rooms with equipment that has been autoclaved, fumigated, or disinfected before re-use. This ensures that the only source of infection is the measured dose of inoculum. A control rabbit in the room used for each dilution is used to assess if inadvertent transmission has occurred. A seven-day period allows RHDV to replicate in the liver if the rabbit is infected [7,53,54,55]. Antibodies against RHDV develop between 5 and 7 days post-inoculation [56] However, seven days post-inoculation, RHDV RNA is still readily detected in the liver of surviving infected rabbits [11,56,57].

*Definition of infection*

RHDV infection, or the lack thereof, is established by molecular tests of liver samples from challenged rabbits. Infected rabbits have a high concentration of RHDV; therefore, sample collection needs to be conducted in a manner that minimises the risk of cross-contamination. If viral replication has occurred, this is identified by increased amounts of viral RNA, which is indicated by a lower Ct value in the RT-qPCR assay compared to the inoculum. Non-infected rabbits had higher Cts when compared to the inoculum. This prevents the misclassification of non-infected rabbits with high Ct values, which may result from residual viral RNA from the inoculum.

*Data analysis*

There were high levels of agreement between the RM, DB, and SK methods, along with probit regression. However, it must be acknowledged that the three interpolation analyses share a common bias [16]. These interpolative methods bias RID_50_ towards the middle of the dose range [16,17,22]. This explains the differences observed between the estimates of the interpolative methods and probit regression, such as in E1, E4, E18, and E22 (Table 1). Nonetheless, there was a good level of agreement between the calculation approaches (Figure 2), which is consistent with previous comparisons and supports the use of an alternative analysis to calculate RID_50_ [16,22,23,58].

A calculation approach that includes an uncertainty estimate is beneficial for assessing the precision of the measurement. Although the RM method is frequently used to calculate RID_50_, this approach cannot provide an uncertainty estimate [17]. In contrast, the improved SK and probit regression methods provide a statistical measure of precision [16]. These estimates assume the homogeneity of the rabbits challenged in the bioassay, a condition that is met by rabbit selection and random assignment to treatment groups [16]. Similar to TCID_50_ calculations in cell cultures, the 95% confidence intervals for RID_50_ are wider with the probit method than with the improved SK method (Table 1) [58]. For instance, the probit PL method gave infinite 95% CI (Table 1). A regression that cannot resolve the confidence limits indicates that the experiment has not included the relevant dilution range to calculate the 50% end point. Similarly, if the choice of dilutions remains entirely above or below the 50% infectious dose, such as in E6 and E26, the RM and DB methods cannot interpolate the endpoint (Table 1). When interpolation is not possible, repeat titrations are necessary to obtain an accurate RID_50_. The improved SK or probit regression methods can inform the choice of dilution factors for the subsequent bioassay based on the confidence limit. For example, the online calculator for probit regression (https://virus-shiny-data.shinyapps.io/RID50/) will identify the dilutions predicted to find an endpoint and refine the sample size and dose range required for the desired level of precision before challenging animals.

## 5. Conclusions

This study outlines a standardised method for the bioassay of RHDV preparations and the key details for obtaining an accurate measure of the concentration of infectious virus expressed as RID_50_. The standardisation of bioassay conditions with consideration of the important aspects of RHDV infection of rabbits provides a reliable measure of RID_50_ that facilitates comparison of virulence, pathogenicity, and vaccine efficacy both within and between scientific institutions.

## Figures and Tables

**Table 1:** The log_10_ titre determined for 26 different preparations of RHDV expressed as median rabbit infectious dose (RID_50_) per mL using four calculation methods: Reed–Muench, Dragstedt–Behrens, improved Spearman–Kärber, and probit analysis. The 95% confidence intervals (CI) for the improved Spearman–Kärber and probit estimates are provided. Experiments where a 50% endpoint could not be determined are noted as “Not available; NA”. Where the upper or lower bounds of the CI are infinite, this has been indicated as “∞”.

Experiment	Reed–Muench	Dragstedt–Behrens	Spearman–Kärber	95% CI	Probit	95% CI
E1	3.60	3.62	3.67	2.84–4.50	4.01	−∞ to +∞
E2	4.97	4.93	5.00	4.60–5.40	5.19	4.65–5.47
E3	4.20	4.21	4.37	3.79–4.94	4.23	1.85–5.06
E4	4.63	4.58	4.51	3.81–5.22	6.35	−∞ to +∞
E5	4.00	4.00	4.00	3.55–4.45	4.00	3.60–5.43
E6	NA	NA	4.17	3.75–4.59	4.07	3.76 to +∞
E7	4.38	4.38	4.33	3.65–5.02	4.46	3.72–6.51
E8	6.09	6.20	6.08	5.25–6.92	5.35	4.90–7.01
E9	3.00	3.00	3.00	2.55–3.45	3.00	2.60–3.40
E10	5.83	5.83	5.83	5.07–6.59	5.96	4.84–8.84
E11	3.50	3.50	3.50	2.90–4.10	3.50	2.94–4.06
E12	6.50	6.50	6.50	5.90–7.10	6.82	5.93 to +∞
E13	3.50	3.50	3.50	3.03–3.97	3.50	2.90–3.96
E14	3.50	3.50	3.50	2.90–4.10	3.45	1.99–4.06
E15	3.33	3.36	3.33	2.80–3.87	3.31	2.05–3.84
E16	6.47	6.48	6.47	5.95–6.99	6.47	5.92–7.11
E17	6.43	6.45	6.50	5.80–7.20	6.53	5.65–7.38
E18	3.57	3.59	3.67	2.92–4.41	2.69	−∞ to +∞
E19	3.71	3.69	3.67	3.05–4.28	3.61	2.16–4.31
E20	4.71	4.68	4.70	4.07–5.33	4.76	4.12–7.93
E21	3.13	3.17	3.10	2.61–3.59	3.04	2.12–3.51
E22	6.50	6.45	6.33	5.56–7.11	9.43	−∞ to +∞
E23	4.00	4.00	4.00	3.55–4.45	4.00	3.60–4.40
E24	4.00	4.00	4.00	3.55–4.45	4.00	3.60–4.40
E25	3.70	3.70	3.83	3.20–4.47	3.78	−∞ to +∞
E26	NA	NA	7.33	7.00–7.67	7.17	6.95 to +∞

**Figure A1:**
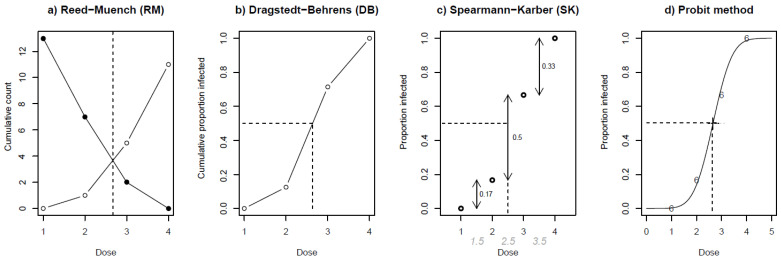
Depiction of the four approaches used to calculate the median infectious dose of an inoculum of rabbit haemorrhagic disease virus, based on a hypothetical bioassay using four doses with none (0/6), one (1/6), four (4/6), and six (6/6) rabbits infected from a total of six rabbits per dilution. The dashed lines on each plot visualise each method’s estimated 50% endpoint. Plots (**a**,**b**) demonstrate the calculation of the 50% endpoint via cumulative counts and proportions, as used in the Reed–Muench (RM) and Dragstedt–Behrens (DB) methods, respectively. To interpolate the 50% endpoint from the RM, two curves are plotted using the cumulative counts of infected (white) and non-infected (black) rabbits. Plot (**c**) shows the Spearman–Kärber (SK) method, which calculates the weighted mean of the doses based on observed proportions. Plot (**d**) depicts the Probit method, which fits a sigmoid curve to the dose–response data to estimate the 50% endpoint.

**Figure A2:**
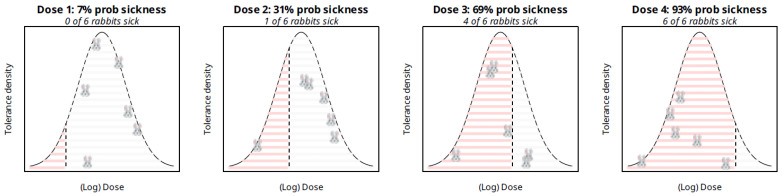
Depiction of the tolerance distribution based on a hypothetical bioassay using four log_10_ doses with 0, 1, 4, and 6 rabbits infected (as plotted in the red–lined area under the curve) from a total of 6 replicates challenged per dilution. The dose–response relationship from this tolerance distribution can be fitted to a sigmoid curve to estimate the 50% endpoint for the probit method.

## Data Availability

The original contributions presented in this study are included in the article/Supplementary Material. Further inquiries can be directed to the corresponding author.

## Supplementary Materials

The following supporting information can be downloaded at: https://www.mdpi.com/article/10.3390/v17050609/s1, Figure S1: Estimates of the median rabbit infectious dose (RID_50_).

## Abbreviations

The following abbreviations are used in this manuscript:

Ct	Cycle-threshold value
DB	Dragstedt–Behrens
G	Genogroup
ICC	Intra-class correlation coefficient
RHDV	Rabbit haemorrhagic disease virus
RID_50_	Median rabbit infectious dose
RM	Reed–Muench
RT-qPCR	Reverse transcription real-time PCR
SK	Spearman–Kärber
TCID_50_	Median tissue culture infectious dose

## Appendix A

This example has been provided to demonstrate the four approaches to calculate RID_50_. An inoculum of RHDV was prepared in a ten-fold dilution series with no infected rabbits (0/6) at the first dose, one infected rabbit at the second dose (1/6), four infected rabbits at the third dose (4/6), and finally all six infected rabbits at the fourth dose (6/6). The RM [18], DB [19], and SK methods [20,21] are interpolative methods based on the formation of cumulative sums or proportions (Table A1). These methods follow the assumption that an infected animal will also be infected at higher doses and that an animal that is not infected will not be infected at lower doses (higher dilutions). The RM method uses the cumulative counts (Figure A1a). The DB method uses cumulative proportions (Figure A1b). Finally, the SK method calculates each dose’s weighted mean infection proportion (Figure A1c).

**Table A1:** An example of the values obtained from the bioassay to determine the median rabbit infectious dose of a hypothetical inoculum of rabbit haemorrhagic disease virus. The counts of infected and non-infected rabbits and cumulative proportions are presented as the preliminary steps for applying the Reed–Muench and Dragstedt–Behrens methods to estimate the 50% endpoint.

Dose	log_10_ Dilution Factor	Counts				Cumulative			
		Infected	Non- Infected	Total	Proportion Infected (Infected/Total)	Infected	Non- Infected	Total	Proportion Infected (Infected/Total)
1	−4	0	6	6	0	0	13	13	0
2	−3	1	5	6	0.167	1	7	8	0.125
3	−2	4	2	6	0.667	5	2	7	0.714
4	−1	6	0	6	1	11	0	11	1

As a curve-fitting method, probit regression does not rely on an assumption of successive infection probability and accounts for the tolerance distribution for individual rabbits. To estimate RID_50,_ a normally distributed “tolerance distribution” is assumed. That is, each rabbit can ‘tolerate’ a specific dose of RHDV before becoming infected (Figure A2) [17,59]. When these tolerances are normally distributed across a hypothetical population of rabbits, the resulting dose–response relationship can be fitted in a sigmoid regression curve (Figure A1d).

The probit regression curve can be fitted using maximum likelihood, and this can be analysed as a generalised linear model (GLM) with a binomial distribution and a probit link function. The probit model estimates the relationship between the dilution ($d_{i}$) of the inoculum and the probability of infection ($p_{i}$) and can be summarised as follows:

(A1) $$p_{i\;}=\phi (\alpha +\beta d_{i})$$

where Φ is the cumulative normal density function and α and β are the two parameters to be estimated. Once estimates α and β are obtained, the infective titre of an inoculum at which 50% of rabbits become infected (RID_50_) can be estimated as −α/β, and the standard error can be extracted from the model.

The 95% confidence interval can be determined using a profile likelihood function (PL). To estimate the PL, the probit model (A1) can also be rewritten as a function of the RID_50_ (A2).

(A2) $$p_{i\;}=\phi (\beta \left(d_{i}-{RID}_{50}\right))$$

Using Equation (2), a 95% profile likelihood confidence interval can be defined as the set of all RID_50_ values satisfying the following:

(A3) $$l\left(\hat{\beta }({RID}_{50}),{RID}_{50}\right)\geq \;l(\hat{\beta ,}\hat{{RID}_{50}})-0.5\times \chi_{1;\;0.95}^{2}$$

where ℓ is the log-likelihood function, $\hat{\beta }$ and $\hat{{RID}_{50}}$ are the maximum likelihood estimates, $\hat{\beta }({RID}_{50})$ is the maximum likelihood estimate of β given RID_50_, and $\chi_{1;\;0.95}^{2}$ is the 95% point of a $\chi^{2}$ distribution with 1 degree of freedom. The 95% confidence interval will be defined by the minimum and maximum values where Equation (A3) is satisfied.
