# Supplementary material for: A Standardised Method to Quantify the Infectious Titre of Rabbit Haemorrhagic Disease Virus
Source: Viruses. 2025 Apr 24;17(5):609. doi: 10.3390/v17050609 (PMC12115570; doi:10.3390/v17050609)
Supplement: Supplementary file 1 [file viruses-17-00609-s001.zip › viruses-3533118-supplementary.pdf]

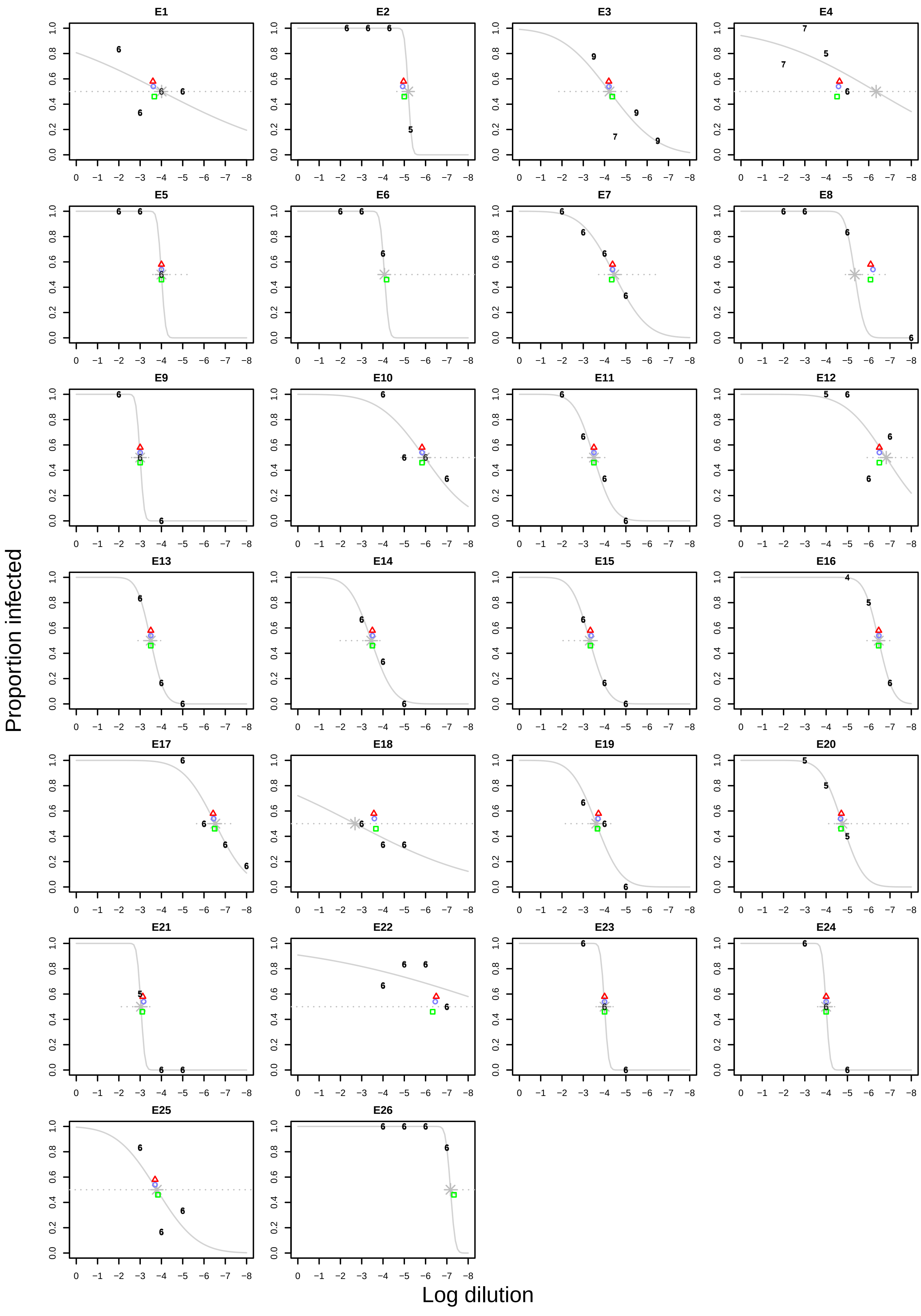
E1
E2
E3
E4
E5
E6
E7
E8
E9
E10
E11
E12
E13
E14
E15
E16
E17
E18
E19
E20
E21
E22
E23
E24
E25
E26
Proportion infected
Log dilution

Supplementary Figure S1. Estimates of the median rabbit infectious dose (RID_{50}) derived from 26 experiments using four methods of calculation: Reed-Muench (red triangles), Dragstedt-Behrens (blue circles), Spearman-Karber (green squares), and probit analysis (grey stars). Each panel depicts results from a single experiment, highlighting the similarities in the RID_{50} between all calculation methods. The number of rabbits inoculated at each dose (log_{10} dilution) is plotted against the proportion infected within each group. The 95% confidence intervals (95% CI) for the probit method are presented as horizontal grey-dashed lines.
